# Genome-wide analysis of ATP binding cassette (ABC) transporters in tomato

**DOI:** 10.1371/journal.pone.0200854

**Published:** 2018-07-26

**Authors:** Peter Amoako Ofori, Ayaka Mizuno, Mami Suzuki, Enrico Martinoia, Stefan Reuscher, Koh Aoki, Daisuke Shibata, Shungo Otagaki, Shogo Matsumoto, Katsuhiro Shiratake

**Affiliations:** 1 Graduate School of Bioagricultural Sciences, Nagoya University, Nagoya, Japan; 2 Department of Plant and Microbial Biology, University of Zurich, Zurich, Switzerland; 3 Graduate School of Life and Environmental Sciences, Osaka Prefecture University, Sakai, Japan; 4 Kazusa DNA Research Institute, Kisarazu, Japan; Meiji Daigaku - Ikuta Campus, JAPAN

## Abstract

ATP binding cassette (ABC) transporters are proteins that actively mediate the transport of a wide range of molecules, such as organic acids, metal ions, phytohormones and secondary metabolites. Therefore, ABC transporters must play indispensable roles in growth and development of tomato, including fruit development. Most ABC transporters have transmembrane domains (TMDs) and belong to the ABC protein family, which includes not only ABC transporters but also soluble ABC proteins lacking TMDs. In this study, we performed a genome-wide identification and expression analysis of genes encoding ABC proteins in tomato (*Solanum lycopersicum*), which is a valuable horticultural crop and a model plant for studying fleshy fruits. In the tomato genome, a total of 154 genes putatively encoding ABC transporters, including 9 ABCAs, 29 ABCBs, 26 ABCCs, 2 ABCDs, 2 ABCEs, 6 ABCFs, 70 ABCGs and 10 ABCIs, were identified. Gene expression data from the eFP Browser and reverse transcription-semi-quantitative PCR analysis revealed their tissue-specific and development-specific expression profiles. This work suggests physiological roles of ABC transporters in tomato and provides fundamental information for future studies of ABC transporters not only in tomato but also in other Solanaceae species.

## Introduction

ATP binding cassette (ABC) proteins are proteins harboring an ATP binding domain, called nucleotide binding domain or fold (NBD/NBF), which contains highly conserved motifs, such as the Walker A and Walker B motifs, the ABC signature, the H loop and the Q loop [1]. ABC proteins are universally found in all organisms, including fungi, plants and animals [2]. Some members of the ABC proteins are soluble proteins and do not contain any transmembrane domain (TMD). The ABC proteins harboring TMDs are called ABC transporters and function as ATP-driven primary transporters for active transport of various molecules [3]. A typical functional ABC transporter contains 2 NBDs and 2 TMDs. The two NBDs synergistically bind and hydrolyze ATP to generate energy, which eventually causes conformational changes in the TMDs to create a pore for substrate transport, whiles the TMDs serve as a pathway for unidirectional transport of the substrate [1]. ABC transporters harboring two TMDs and two NBDs are called full-size ABC transporters. On the other hand, ABC transporters harboring only one TMD and one NBD are called half-size. ABC transporters encoded by four genes, two for TMDs and two for NBDs are so-called quarter-size ABC transporters [3,4].

ABC transporters are grouped into eight subfamilies, namely ABCA to ABCI. Plants do not have any ABCH subfamily. Generally, plants possess twice as many as ABC transporters as not in animals. It is assumed that this is due to the sessile nature of plants for growing under various biotic and abiotic stresses [5]. ABC transporters of plants are engaged in numerous functions, including secondary metabolite transport [6,7], heavy metal detoxification [8], antibiotic transport [9] and phytohormone transport [10,11]. ABC transporter counterparts in animal are also shown to function as ion channels, channel regulators [12,13] and in protein targeting [14].

A genome-wide analysis is the comprehensive identification of all genes of the respective family including their family members and organization of their information. This approach provides essential information, such as evolutionary history, diversity and relationship among genes and proteins, which serves as useful fundamental resources for further investigations. Genome-wide analyses of ABC transporters in Arabidopsis [15], rice [16], maize [17], *Lotus japonicus* [18], grape [19], pineapple [20], and *Hevea brasiliensis* [4] have already been performed. Whereas little is known about ABC transporters in Solanaceae, including tomato.

Tomato is an important vegetable crop and is often used as a model plant for studying developmental physiology of fleshy fruits recently. The advantages of tomato in research are the availability of its high quality whole genome sequencing data (Sol Genomics Network (SGN), https://solgenomics.net/) [21], expressed sequence tag (EST) database (TomatEST, http://biosrv.cab.unina.it/tomatestdb/transcript_browser.html) [22] and full-length cDNA resources (TOMATOMICS: http://plantomics.mind.meiji.ac.jp/tomatomics/) [23,24]. Transcriptome databases at Tomato eFP Browser (http://bar.utoronto.ca/efp_tomato/cgi-bin/efpWeb.cgi) [25,26] and SGN-TEA (http://tea.solgenomics.net/) [27] and metabolome database at MoTo DB (http://www.transplantdb.eu/node/1843) [28] are also available for tomato. Micro-Tom is a dwarf tomato variety and an excellent tool for genetic and physiological studies of fruit development and physiology, because of its small size and short lifecycle [29].

In this study, a genome wide analysis was performed to provide information of ABC proteins in tomato. A total of 154 genes putatively encoding ABC proteins were identified in tomato genome. Among these ABC proteins, 47 proteins are soluble ABC proteins lacking any TMDs, while 107 proteins contain TMDs and they are considered to function as ABC transporters. Phylogenetic analysis revealed the evolutionary relationships of tomato ABC proteins. In addition, protein structure, in silico and reverse transcription-semi-quantitative PCR gene expression analyses were performed to provide fundamental information for further ABC protein studies not only in tomato but also in other Solanaceae species.

## Materials and methods

### Identification of ABC proteins in tomato

The BLAST tool of Sol Genomics Network (SGN, http://www.solgenomics.net/) [21] was used for genome-wide identification of genes encoding ABC proteins in tomato. Known ABC proteins of tomato reported by Andolfo et al. [30] and some members of the Arabidopsis ABC subfamilies [15] were used as queries for BLAST search in the tomato genome (SL3.0 and ITAG3.10) [26]. Identified proteins with at least 30% similarity to the query sequence or E-value less than E-20 were selected. Presence of ABC signature, Walker A and Walker B motifs was confirmed by using the Conserved Domain Database of NCBI (https://www.ncbi.nlm.nih.gov/cdd/) [31]. The predicted genes encoding ABC proteins from SL3.0 of SGN were confirmed by comparing with another tomato genome database TMCSv1.2.1 from TOMATOMICS (http://plantomics.mind.meiji.ac.jp/tomatomics/download.php) [23,24].

#### Phylogenetic, in silico gene expression and protein structure analyses

Phylogenetic analysis was conducted to classify the identified ABC proteins into their respective subfamilies. Entire protein sequences of ABC proteins were aligned using the multiple sequence alignment tool of ClustalW program (http://www.genome.jp/tools/clustalw/) [32] and subjected to cluster analysis by the distance with the neighbor-joining method using MEGA6.06 software (Molecular Evolutionary Genetics Analysis, https://www.megasoftware.net/) [33]. Gene expression data of ABC proteins in various tomato tissues were obtained from the Tomato eFP Browser (http://bar.utoronto.ca/efp_tomato/cgi-bin/efpWeb.cgi) [25,26]. The Pfam web server (http://pfam.xfam.org/) [34] was used to characterize the topology of ABC proteins comprising TMD and NBD.

#### Plant materials

Tomato (*Solanum lycopersicum*) 'Micro-Tom' was used for gene expression analysis. The Micro-Tom strain used in this study was obtained from the National Bioresource Project (NBRP)-Tomato (http://tomato.nbrp.jp/browseSearchEn.html) with an accession number TOMJPF00001. Plants were grown in growth chamber (Biotron LPH-350S, NK Systems) adjusted to 25°C, 16 h light/8 h dark period and 60% relative humidity. Tap water was supplied twice a week. Half concentration of Otsuka liquid fertilizer (Otsuka Chemicals Co., Ltd.) was applied weekly. Young and mature leaves, root, stem, flower, developing fruit tissues at 3, 7, 14, 21, 28 days after pollination (DAP), breaker, orange and red stages were sampled, frozen in liquid nitrogen and stored at -80°C.

#### RNA extraction and RT-semi-quantitative PCR (RT-sqPCR) expression analysis

Extraction of total RNA from developing fruits at 14 and 21 DAP was performed using the RNA Suisui-R kit (Rizo). RNA of other tissues was isolated using TRIzol reagent (Life Technologies). PrimeScript RT reagent kit (Takara) was used to synthesize the cDNA. RT-sqPCR was conducted using SYBR Premix Ex Taq kit (Takara) and the ubiquitin gene, *SlUBQ* (*Solyc01g056940*) was used as an internal control. Primer sequences and PCR conditions are shown in S1 Table.

## Results and discussion

### Genome-wide identification of ABC proteins in tomato

**Fig 1:**
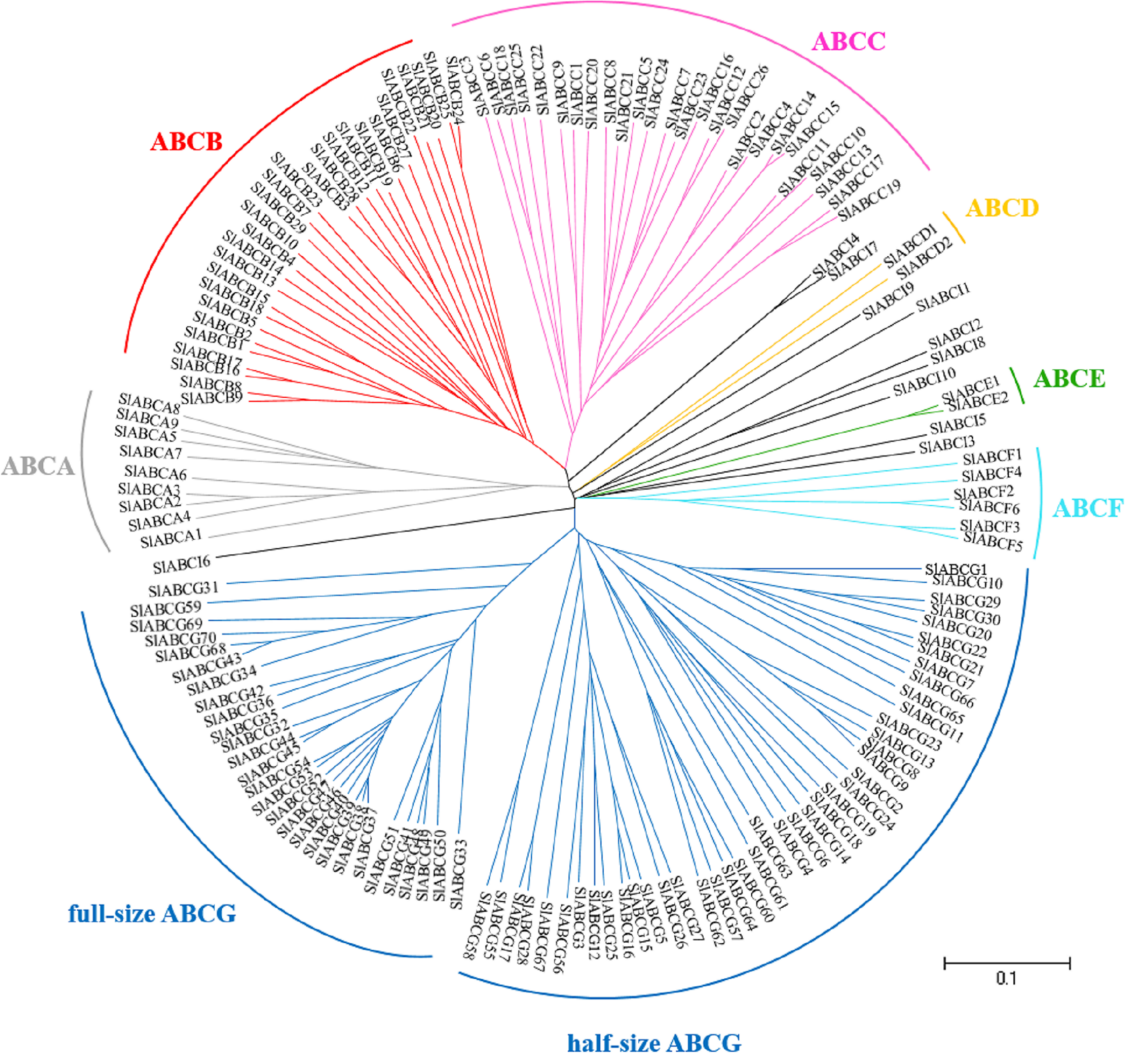
Phylogenetic tree of tomato ABC proteins. The 154 ABC proteins identified were subjected to phylogenetic analysis. Subfamily names (ABCA-I, except ABCH) correspond to the mammalian ABC transporter nomenclature. Tomato ABC proteins not clustered in ABCA-ABCG subfamilies are ABCIs. The scale indicated in the figure shows 10% divergence between protein sequences.

To clarify the gene family of ABC proteins in tomato, BLAST search on tomato genome database Sol Genomics Network (SGN, http://www.solgenomics.net/) [21] was performed. We searched all the tomato ABC proteins using SL3.0 of SGN database. As a result, 154 genes potentially encoding ABC proteins were found (Table 1). Phylogenetic analysis of the tomato ABC proteins was performed and the obtained phylogenetic tree is shown in Fig 1.

**Table 1:** Inventory of tomato ABC proteins with their in silico gene expression profiles.

Sub- family	Gene name	Locus	Size (AA)	Best hit EST	Topology	Old name	Expression										Abs value
							L	R	B	F	F1	F2	F3	M	Bk	Rd	
**ABCA**	**SlABCA1**	***Solyc04g015970*.*2***	**1,910**	**SGN-E342230**	**(TMD-NBD)×2**	** **											**26.7**
	**SlABCA2**	***Solyc03g113040*.*2***	**946**	**SGN-E1283433**	**TMD-NBD**	** **											**10.9**
	**SlABCA3**	***Solyc03g113060*.*2***	**945**	**SGN-E1279186**	**TMD-NBD**	** **											**41.1**
	**SlABCA4**	***Solyc06g070920*.*2***	**639**	**SGN-E547444**	**TMD-NBD**	** **											**31.0**
	**SlABCA5**	***Solyc06g070940*.*2***	**944**	**SGN-E373720**	**TMD-NBD**	** **											**21.7**
	**SlABCA6**	***Solyc06g070950*.*1***	**892**	**-**	**TMD-NBD**	** **											**0.85**
	**SlABCA7**	***Solyc06g070960*.*1***	**927**	**-**	**TMD-NBD**	** **											**0.57**
	**SlABCA8**	***Solyc03g113080*.*2***	**359**	**-**	**NBD**	** **											**13.8**
	**SlABCA9**	***Solyc03g113070*.*2***	**577**	**-**	**NBD**	** **											**11.7**
**ABCB**	**SlABCB1**	***Solyc02g071340*.*1***	**1,264**	**-**	**(TMD-NBD)×2**	** **											**0.04**
	**SlABCB2**	***Solyc02g071350*.*2***	**1,264**	**-**	**(TMD-NBD)×2**	** **											**1.89**
	**SlABCB3**	***Solyc02g087410*.*2***	**1,263**	**-**	**(TMD-NBD)×2**	** **											**5.79**
	**SlABCB4**	***Solyc02g087870*.*2***	**1,250**	**SGN-E701700**	**(TMD-NBD)×2**	**SlMDR1**											**26.6**
	**SlABCB5**	***Solyc03g005860*.*2***	**1,260**	**-**	**(TMD-NBD)×2**	** **											**0.14**
	**SlABCB6**	***Solyc03g093650*.*2***	**1,228**	**-**	**(TMD-NBD)×2**	** **											**3.95**
	**SlABCB7**	***Solyc04g010310*.*2***	**1,286**	**SGN-E1249810**	**(TMD-NBD)×2**	** **											**20.7**
	**SlABCB8**	***Solyc06g009280*.*1***	**1,290**	**-**	**(TMD-NBD)×2**	** **											**5.56**
	**SlABCB9**	***Solyc06g009290*.*2***	**1,401**	**SGN-E550470**	**(TMD-NBD)×2**	**SlMDR2**											**47.3**
	**SlABCB10**	***Solyc06g072960*.*1***	**1,029**	**-**	**(TMD-NBD)×2**	** **											**0.02**
	**SlABCB11**	***Solyc07g018130*.*1***	**1,276**	**-**	**(TMD-NBD)×2**	** **											**0.04**
	**SlABCB12**	***Solyc07g064120*.*1***	**1,260**	**-**	**(TMD-NBD)×2**	** **											**0.39**
	**SlABCB13**	***Solyc08g076720*.*2***	**1,258**	**SGN-E228826**	**(TMD-NBD)×2**	** **											**25.8**
	**SlABCB14**	***Solyc09g008240*.*2***	**1,315**	**SGN-E315464**	**(TMD-NBD)×2**	** **											**52.1**
	**SlABCB15**	***Solyc11g067310*.*1***	**1,290**	**-**	**(TMD-NBD)×2**	** **											**0.61**
	**SlABCB16**	***Solyc12g098840*.*1***	**1,281**	**-**	**(TMD-NBD)×2**	** **											**25.4**
	**SlABCB17**	***Solyc12g098870*.*1***	**1,313**	**-**	**(TMD-NBD)×2**	** **											**2.89**
	**SlABCB18**	***Solyc11g067300*.*1***	**1,261**	**-**	**TMD-TMD-NBD-TMD-NBD**	** **											**3.16**
	**SlABCB19**	***Solyc05g013890*.*1***	**955**	**-**	**NBD-TMD-NBD**	** **											**0.28**
	**SlABCB20**	***Solyc03g026310*.*2***	**664**	**SGN-E1284789**	**TMD-NBD**	** **											**32.4**
	**SlABCB21**	***Solyc03g114950*.*2***	**639**	**SGN-E746787**	**TMD-NBD**	** **											**57.7**
	**SlABCB22**	***Solyc03g122050*.*1***	**673**	**-**	**TMD-NBD**	** **											**0.20**
	**SlABCB23**	***Solyc03g122070*.*1***	**667**	**-**	**TMD-NBD**	** **											**0.49**
	**SlABCB24**	***Solyc09g009910*.*2***	**640**	**SGN-E1285685**	**TMD-NBD**	** **											**3.52**
	**SlABCB25**	***Solyc09g055350*.*2***	**726**	**SGN-E1270076**	**TMD-NBD**	** **											**28.2**
	**SlABCB26**	***Solyc00g304030*.*1***	**1,081**	**-**	**NBD-TMD**	** **											**0.00**
	**SlABCB27**	***Solyc12g049120*.*1***	**349**	**-**	**NBD**	** **											**0.00**
	**SlABCB28**	***Solyc12g049130*.*1***	**108**	**-**	**NBD**	** **											**0.00**
	**SlABCB29**	***Solyc12g070280*.*1***	**232**	**SGN-E276294**	**NBD**	** **											**53.8**
**ABCC**	**SlABCC1**	***Solyc01g080640*.*2***	**1,499**	**SGN-E701199**	**(TMD-NBD)×2**	** **											**116**
	**SlABCC2**	***Solyc03g007530*.*2***	**1,468**	**SGN-E313421**	**(TMD-NBD)×2**	** **											**15.2**
	**SlABCC3**	***Solyc03g117540*.*2***	**1,482**	**SGN-E230070**	**(TMD-NBD)×2**	** **											**6.79**
	**SlABCC4**	***Solyc06g036490*.*1***	**1,194**	**-**	**(TMD-NBD)×2**	** **											**0.02**
	**SlABCC5**	***Solyc07g065320*.*2***	**1,506**	**SGN-E206721**	**(TMD-NBD)×2**	** **											**8.11**
	**SlABCC6**	***Solyc08g006880*.*2***	**1,627**	**SGN-E303088**	**(TMD-NBD)×2**	**SlMRP2**											**114**
	**SlABCC7**	***Solyc08g081890*.*2***	**1,480**	**SGN-E1281130**	**(TMD-NBD)×2**	** **											**28.6**
	**SlABCC8**	***Solyc09g064440*.*2***	**1,532**	**SGN-E1307571**	**(TMD-NBD)×2**	** **											**23.3**
	**SlABCC9**	***Solyc09g075020*.*2***	**1,514**	**SGN-E345495**	**(TMD-NBD)×2**	** **											**66.0**
	**SlABCC10**	***Solyc10g019270*.*1***	**1,220**	**-**	**(TMD-NBD)×2**	** **											**3.39**
	**SlABCC11**	***Solyc10g024420*.*1***	**1,478**	**SGN-E128420**	**(TMD-NBD)×2**	**SlMRP1**											**312**
	**SlABCC12**	***Solyc12g044820*.*1***	**1,459**	**SGN-E689095**	**(TMD-NBD)×2**	** **											**53.8**
	**SlABCC13**	***Solyc05g014380*.*2***	**1,136**	**SGN-E1256841**	**TMD-NBD-TMD**	**SlMRP3**											**69.1**
	**SlABCC14**	***Solyc00g283010*.*1***	**646**	**-**	**TMD-NBD**	** **											**1.38**
	**SlABCC15**	***Solyc11g065710*.*1***	**773**	**-**	**TMD-NBD**	** **											**1.33**
	**SlABCC16**	***Solyc11g065720*.*1***	**652**	**-**	**TMD-NBD**	** **											**1.65**
	**SlABCC17**	***Solyc12g036150*.*1***	**374**	**SGN-E213562**	**TMD-NBD**	** **											**11.4**
	**SlABCC18**	***Solyc12g036140*.*1***	**486**	**-**	**NBD-TMD**	** **											**13.1**
	**SlABCC19**	***Solyc02g044000*.*1***	**604**	**-**	**NBD**	** **											**14.0**
	**SlABCC20**	***Solyc02g044050*.*1***	**492**	**-**	**NBD**	** **											**7.49**
	**SlABCC21**	***Solyc05g014390*.*2***	**282**	**-**	**NBD**	** **											**83.5**
	**SlABCC22**	***Solyc05g014500*.*1***	**90**	**-**	**NBD**	** **											**22.7**
	**SlABCC23**	***Solyc06g036480*.*1***	**135**	**-**	**NBD**	** **											**0.11**
	**SlABCC24**	***Solyc10g019280*.*1***	**54**	**-**	**NBD**	** **											**0.00**
	**SlABCC25**	***Solyc12g036160*.*1***	**233**	**-**	**NBD**	** **											**22.0**
	**SlABCC26**	***Solyc12g044810*.*1***	**166**	**-**	**NBD**	** **											**16.2**
**ABCD**	**SlABCD1**	***Solyc04g055120*.*2***	**1,345**	**SGN-E707100**	**(TMD-NBD)×2**	** **											**37.9**
	**SlABCD2**	***Solyc12g017420*.*1***	**706**	**SGN-E1282388**	**TMD-NBD**	** **											**27.8**
**ABCE**	**SlABCE1**	***Solyc07g008340*.*2***	**579**	**SGN-E745894**	**NBD-NBD**	** **											**79.6**
	**SlABCE2**	***Solyc08g075360*.*1***	**607**	**-**	**NBD-NBD**	** **											**5.04**
**ABCF**	**SlABCF1**	***Solyc04g051800*.*2***	**696**	**SGN-E738084**	**NBD-NBD**	** **											**296**
	**SlABCF2**	***Solyc06g074940*.*2***	**575**	**SGN-E745759**	**NBD-NBD**	** **											**33.7**
	**SlABCF3**	***Solyc07g008610*.*1***	**696**	**SGN-E1284822**	**NBD-NBD**	** **											**65.7**
	**SlABCF4**	***Solyc08g082850*.*2***	**717**	**SGN-E745898**	**NBD-NBD**	** **											**131**
	**SlABCF5**	***Solyc10g012190*.*1***	**688**	**-**	**NBD-NBD**	** **											**0.12**
	**SlABCF6**	***Solyc11g069090*.*1***	**602**	**SGN-E717588**	**NBD-NBD**	** **											**1,277**
**ABCG**	**SlABCG1**	***Solyc01g006720*.*2***	**725**	**SGN-E539555**	**NBD-TMD**	** **											**34.3**
	**SlABCG2**	***Solyc01g097430*.*2***	**839**	**-**	**NBD-TMD**	** **											**12.7**
	**SlABCG3**	***Solyc01g105450*.*2***	**628**	**SGN-E320849**	**NBD-TMD**	** **											**19.7**
	**SlABCG4**	***Solyc03g007690*.*1***	**598**	**SGN-E711119**	**NBD-TMD**	** **											**42.2**
	**SlABCG5**	***Solyc03g019760*.*2***	**711**	**SGN-E345650**	**NBD-TMD**	** **											**28.8**
	**SlABCG6**	***Solyc03g113690*.*1***	**659**	**-**	**NBD-TMD**	** **											**1.06**
	**SlABCG7**	***Solyc04g006960*.*2***	**676**	**SGN-E205662**	**NBD-TMD**	**SlWBC8**											**4.69**
	**SlABCG8**	***Solyc04g010200*.*1***	**719**	**SGN-E1278817**	**NBD-TMD**	**SlWBC4**											**24.5**
	**SlABCG9**	***Solyc04g010210*.*1***	**715**	**SGN-E1282871**	**NBD-TMD**	**SlWBC5**											**4.28**
	**SlABCG10**	***Solyc04g070970*.*2***	**723**	**SGN-E328516**	**NBD-TMD**	** **											**43.3**
	**SlABCG11**	***Solyc05g008350*.*2***	**711**	**SGN-E730349**	**NBD-TMD**	**SlWBC10**											**43.8**
	**SlABCG12**	***Solyc05g051530*.*2***	**531**	**SGN-E349400**	**NBD-TMD**	**SlWBC7**											**47.9**
	**SlABCG13**	***Solyc05g054890*.*2***	**751**	**SGN-E1255617**	**NBD-TMD**	**SlWBC3**											**6.54**
	**SlABCG14**	***Solyc05g056470*.*1***	**615**	**-**	**NBD-TMD**	** **											**9.74**
	**SlABCG15**	***Solyc06g072090*.*1***	**661**	**-**	**NBD-TMD**	** **											**0.47**
	**SlABCG16**	***Solyc06g072100*.*1***	**716**	**-**	**NBD-TMD**	** **											**0.48**
	**SlABCG17**	***Solyc06g074970*.*1***	**603**	**SGN-E1260065**	**NBD-TMD**	**SlWBC6**											**11.5**
	**SlABCG18**	***Solyc07g053300*.*1***	**609**	**-**	**NBD-TMD**	** **											**1.07**
	**SlABCG19**	***Solyc07g062630*.*1***	**622**	**-**	**NBD-TMD**	** **											**0.28**
	**SlABCG20**	***Solyc07g063400*.*2***	**614**	**-**	**NBD-TMD**	** **											**5.50**
	**SlABCG21**	***Solyc08g005580*.*2***	**656**	**SGN-E211225**	**NBD-TMD**	** **											**4.26**
	**SlABCG22**	***Solyc08g075430*.*2***	**647**	**SGN-E706558**	**NBD-TMD**	**SlWBC2**											**41.2**
	**SlABCG23**	***Solyc09g005970*.*1***	**739**	**SGN-E379457**	**NBD-TMD**	** **											**3.11**
	**SlABCG24**	***Solyc09g098410*.*1***	**730**	**-**	**NBD-TMD**	** **											**0.00**
	**SlABCG25**	***Solyc11g009100*.*1***	**650**	**SGN-E218423**	**NBD-TMD**	** **											**31.3**
	**SlABCG26**	***Solyc11g065350*.*1***	**683**	**-**	**NBD-TMD**	** **											**44.5**
	**SlABCG27**	***Solyc11g065360*.*1***	**689**	**-**	**NBD-TMD**	** **											**5.93**
	**SlABCG28**	***Solyc11g069710*.*1***	**724**	**SGN-E1306745**	**NBD-TMD**	**SlWBC1**											**17.0**
	**SlABCG29**	***Solyc12g013630*.*1***	**629**	**-**	**NBD-TMD**	** **											**12.4**
	**SlABCG30**	***Solyc12g013640*.*1***	**631**	**-**	**NBD-TMD**	** **											**0.23**
	**SlABCG31**	***Solyc12g019620*.*1***	**838**	**SGN-E1245045**	**NBD-TMD**	**SlPDR2**											**1.08**
	**SlABCG32**	***Solyc12g019640*.*1***	**609**	**-**	**NBD-TMD**	** **											**2.16**
	**SlABCG33**	***Solyc01g101070*.*2***	**1,448**	**SGN-E542052**	**(NBD-TMD)×2**	** **											**11.8**
	**SlABCG34**	***Solyc02g081870*.*2***	**1,402**	**-**	**(NBD-TMD)×2**	** **											**0.02**
	**SlABCG35**	***Solyc03g120980*.*2***	**1,501**	**SGN-E128965**	**(NBD-TMD)×2**	** **											**93.8**
	**SlABCG36**	***Solyc05g018510*.*2***	**1,422**	**SGN-E699701**	**(NBD-TMD)×2**	**SlPDR1**											**43.2**
	**SlABCG37**	***Solyc05g053570*.*2***	**1,411**	**-**	**(NBD-TMD)×2**	** **											**10.3**
	**SlABCG38**	***Solyc05g053590*.*2***	**1,413**	**-**	**(NBD-TMD)×2**	** **											**50.1**
	**SlABCG39**	***Solyc05g053600*.*2***	**1,413**	**SGN-E1300502**	**(NBD-TMD)×2**	** **											**16.6**
	**SlABCG40**	***Solyc05g053610*.*2***	**1,426**	**SGN-E357332**	**(NBD-TMD)×2**	** **											**174**
	**SlABCG41**	***Solyc05g055330*.*2***	**1,479**	**-**	**(NBD-TMD)×2**	** **											**18.5**
	**SlABCG42**	***Solyc06g065670*.*2***	**1,409**	**SGN-E546084**	**(NBD-TMD)×2**	** **											**12.1**
	**SlABCG43**	***Solyc06g076930*.*1***	**1,426**	**SGN-E243451**	**(NBD-TMD)×2**	** **											**20.7**
	**SlABCG44**	***Solyc08g067610*.*2***	**1,455**	**SGN-E1249186**	**(NBD-TMD)×2**	** **											**40.8**
	**SlABCG45**	***Solyc08g067620*.*2***	**1,454**	**-**	**(NBD-TMD)×2**	** **											**18.5**
	**SlABCG46**	***Solyc09g091660*.*2***	**1,441**	**SGN-E541199**	**(NBD-TMD)×2**	** **											**80.6**
	**SlABCG47**	***Solyc09g091670*.*2***	**1,429**	**SGN-E356859**	**(NBD-TMD)×2**	** **											**16.1**
	**SlABCG48**	***Solyc11g007280*.*1***	**1,469**	**-**	**(NBD-TMD)×2**	** **											**0.03**
	**SlABCG49**	***Solyc11g007290*.*1***	**1,468**	**-**	**(NBD-TMD)×2**	** **											**0.22**
	**SlABCG50**	***Solyc11g007300*.*1***	**1,465**	**-**	**(NBD-TMD)×2**	** **											**0.01**
	**SlABCG51**	***Solyc11g067000*.*1***	**1,464**	**-**	**(NBD-TMD)×2**	** **											**9.49**
	**SlABCG52**	***Solyc12g098210*.*1***	**1,426**	**-**	**(NBD-TMD)×2**	** **											**0.63**
	**SlABCG53**	***Solyc12g100180*.*1***	**1,436**	**SGN-E546066**	**(NBD-TMD)×2**	** **											**57.2**
	**SlABCG54**	***Solyc12g100190*.*1***	**1,429**	**-**	**(NBD-TMD)×2**	** **											**13.5**
	**SlABCG55**	***Solyc00g233480*.*1***	**184**	**-**	**NBD**	** **											**45.6**
	**SlABCG56**	***Solyc01g105400*.*2***	**117**	**SGN-E218425**	**NBD**	** **											**0.75**
	**SlABCG57**	***Solyc04g025170*.*2***	**1,021**	**SGN-E286554**	**NBD**	** **											**16.6**
	**SlABCG58**	***Solyc05g051540*.*1***	**131**	**-**	**NBD**	** **											**12.6**
	**SlABCG59**	***Solyc06g036240*.*1***	**641**	**-**	**NBD**	** **											**0.59**
	**SlABCG60**	***Solyc06g075020*.*2***	**1,095**	**-**	**NBD**	** **											**2.26**
	**SlABCG61**	***Solyc07g065770*.*2***	**227**	**SGN-E327102**	**NBD**	** **											**6.66**
	**SlABCG62**	***Solyc09g008000*.*2***	**1,092**	**SGN-E330243**	**NBD**	** **											**9.86**
	**SlABCG63**	***Solyc11g018690*.*1***	**343**	**SGN-E1293717**	**NBD**	**SlWBC9, 11**											**18.7**
	**SlABCG64**	***Solyc11g069820*.*1***	**1,094**	**-**	**NBD**	** **											**2.63**
	**SlABCG65**	***Solyc07g065780*.*1***	**446**	**-**	**NBD**	** **											**15.2**
	**SlABCG66**	***Solyc11g018680*.*1***	**291**	**SGN-E717727**	**NBD**	** **											**19.9**
	**SlABCG67**	***Solyc00g164680*.*1***	**491**	**-**	**NBD**	** **											**28.9**
	**SlABCG68**	***Solyc02g055530*.*2***	**59**	**-**	**NBD**	** **											**39.4**
	**SlABCG69**	***Solyc04g076170*.*1***	**190**	**-**	**NBD**	** **											**18.9**
	**SlABCG70**	***Solyc09g042280*.*1***	**112**	**-**	**NBD**	** **											**7.20**
**ABCI**	**SlABCI1**	***Solyc00g304030*.*1***	**1,081**	**-**	**NBD**	** **											**0.00**
	**SlABCI2**	***Solyc01g100850*.*2***	**329**	**SGN-E1301393**	**NBD**	** **											**52.8**
	**SlABCI3**	***Solyc02g068180*.*2***	**275**	**SGN-E1307012**	**NBD**	** **											**22.3**
	**SlABCI4**	***Solyc03g117810*.*2***	**264**	**SGN-E1270799**	**NBD**	** **											**105**
	**SlABCI5**	***Solyc04g056650*.*2***	**351**	**SGN-E700042**	**NBD**	** **											**24.1**
	**SlABCI6**	***Solyc06g048540*.*2***	**313**	**SGN-E720007**	**NBD**	** **											**130**
	**SlABCI7**	***Solyc06g068600*.*2***	**186**	**-**	**NBD**	** **											**116**
	**SlABCI8**	***Solyc09g066470*.*2***	**287**	**SGN-E321321**	**NBD**	** **											**79.0**
	**SlABCI9**	***Solyc11g069260*.*1***	**261**	**SGN-E302237**	**NBD**	** **											**19.4**
	**SlABCI10**	***Solyc12g010220*.*1***	**230**	**SGN-E203090**	**NBD**	** **											**8.15**

The best hit ESTs were found by blasting from SGN web server (https://solgenomics.net/). Pfam web server (http://pfam.xfam.org/) was used to identify the conserved domains (topology); NBD: nucleotide binding domain (ATP binding cassette domain); TMD: transmembrane domain. Gene expression profile data in various tomato organs and tissues was obtained from Tomato eFP Browser (http://bar.utoronto.ca/efp_tomato/cgi-bin/efpWeb.cgi). The gene expression levels (low to high) are indicated by the light to deep red color shades. L: leaf; R: root; bud; F: flower; F1: 1cm fruit; F2: 2cm fruit; F3: 3cm fruit; M: mature green; Bk: breaker; Rd: 10 days after breaker; Abs value: RPKM value of maximum gene expression level in various tomato organs and tissues for each gene.

In a previous study, Andolfo et al. [30] identified 180 ABC proteins in the tomato genome, whiles we found 154 ABC proteins. So we compared non-overlapping candidates between our study and Andolfo et al. [30] (S2 Table). In this study, 3 non-overlapping putative tomato ABC proteins were identified whereas 29 ABC proteins were identified only in Andolfo et al. [30] (S2 Table). All the 3 ABC proteins identified in this study have NBDs. On the other hand, the 29 ABC proteins found only in Andolfo et al. [30] have no NBD. Thus, we concluded that the 29 candidates without NBD in Andolfo et al. [30] are not ABC proteins and may be mispredicted. Therefore, we did not include them in our list (Table 1).

In addition, since some of the genes may not be computationally annotated in SL3.0 of SGN database, we confirmed the gene prediction of SL3.0 by comparing this database with another tomato genome database, TMCSv1.2.1 from TOAMTOMICS [23,24] (S3 Table). As a result, no new tomato ABC proteins were found in TMCSv1.2.1. However, corresponding genes of *SlABCA8*, *SlABCC22*, *SlABCC24* and *SlABCG68* identified in SL3.0 were not identified in TMCSv1.2.1 (S3 Table). The tomato eFP browser showed gene expression data for *SlABCA8*, *SlABCC22* and *SlABCG68* (Table 1), suggesting that these genes may be functional genes. On the other hand, the tomato eFP browser showed no gene expression for *SlABCC24* (Table 1), suggesting that *SlABCC24* may have been mispredicted. The SL3.0 tomato genome database suggests only one transcript for one locus, on the other hand, TMCSv1.2.1 suggests several splicing variants for one locus (S3 Table).

Wider research coverage on ABC transporters has caused emergence of several naming schemes. In most cases, they were named based on the mutant characteristics. This eventually resulted in assigning different names to the same subfamily or selected members with common characteristics [35]. To conform to plant and animal ABC communities, the Human Genome Organization (HUGO) nomenclature system [35] was adopted to designate all putatively ABC proteins into their diverse subfamilies (Fig 1). A unified ABC nomenclature proposed by Verrier et al. [35] was also used to assign ABCA-ABCG and ABCI to all the eight subfamilies (Table 1).

The 154 ABC proteins identified in the tomato genome were grouped into 9 ABCAs, 29 ABCBs, 26 ABCCs, 2 ABCDs, 2 ABCEs, 6 ABCFs, 70 ABCGs and 10 ABCIs (Table 1, Fig 1). The most abundant subfamily members were ABCB, ABCC and ABCG; while ABCD and ABCE were the least abundant. This characteristic is similar to the distribution of ABC proteins in human [36] and other plants, such as Arabidopsis [15], rice [16], *L*. *japonica* [18] and *H*. *brasiliensis* [4]. At least one EST in the SGN database (http://www.solgenomics.net/) [21] was found for 78 genes. The reason for the absence of ESTs for the 69 genes could be that they are either expressed only under certain conditions or in specific cell types. Alternatively, they could represent pseudogenes as suggested in genome-wide analysis of tomato aquaporins and sugar transporters [37,38].

A typical full-size of ABC protein has >1,200 amino acid residues [39]. The sizes of the 154 ABC proteins of tomato ranged from 50 to over 1,910 amino acid residues, although all of them possess at least one NBD as shown in Table 1. Some of the tomato ABC proteins with shorter sequences might be pseudogene or misannotation as suggested in the genome-wide analysis of tomato aquaporins and sugar transporters [37,38]. Among the 154 tomato ABC proteins, 47 members are lacking a TMD and are considered as soluble ABC proteins (Table 1). On the other hand, the other 107 members possess TMDs and are considered as ABC transporters.

One of the unique features of ABC proteins is their topological diversity. Structural orientation and conserved domains for each protein predicted by the Pfam web server is shown in Table 1. Fifty-four ABC proteins are full-size proteins possessing (TMD-NBD)x2. Among these members, 32 exhibit a forward, while 22 have a reverse topology orientations. Fifty-three ABC proteins were half-size having (TMD-NBD)x1 or (NBD-TMD)x1. Among the half-size ABC proteins, 18 exhibit a forward and 35 a reverse domain orientations. Forty-seven ABC proteins are considered as quarter-size ABC transporter proteins. SlABCB19 and SlABCC13 were uniquely characterized with NBD-TMD-NBD and TMD-NBD-TMD orientations, respectively. Similar topological patterns were reported in ABC proteins of rice [16], maize [17] and *L*. *japonica* [18]. Such characteristics might have resulted from gene duplication or evolved to render specicific physiological functions [40].

### The tomato ABC protein subfamilies

#### ABCA subfamily

**Fig 2:**
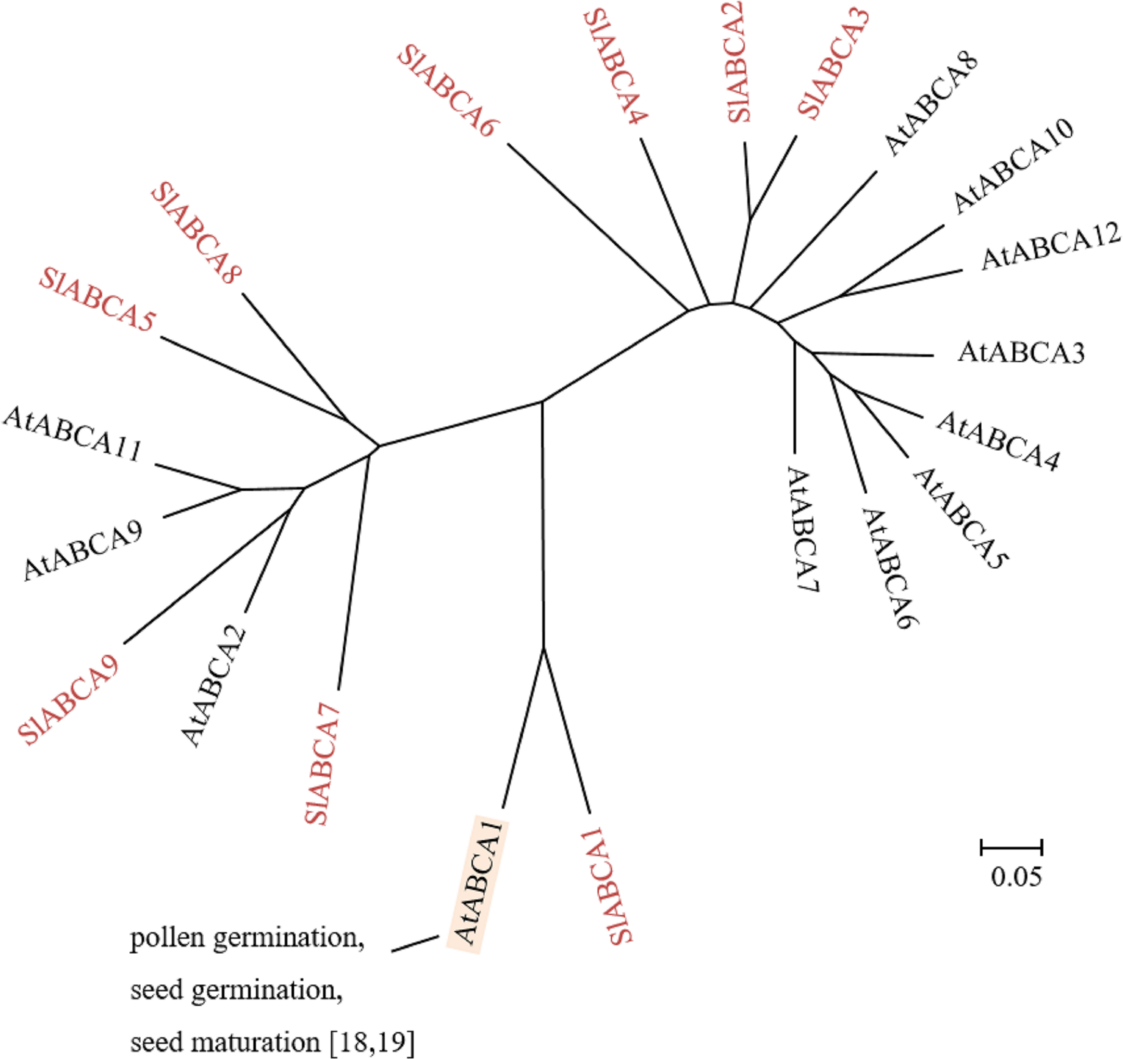
Phylogenetic tree of plant ABCA subfamily. ABCAs of tomato and Arabidopsis were subjected to phylogenetic analysis. Tomato ABCAs are shown in red. Physiological functions and references are indicated. The scale indicated in the figure shows 5% divergence between protein sequences.

The plant ABCA subfamily is made up of one full-size ABCA and several half-size ABCAs. In Arabidopsis, AtABCA1, also known as ABC one homologue (AOH), is the only full-size ABCA protein and is the largest ABC protein, consisting of 1,882 amino acid residues [15,16]. The remaining are half-size ABCAs are also called ABC two homologues (ATH). In tomato genome, 9 members of the ABCA subfamily were found (Table 1, Fig 2). SlABCA1 was the only full-size ABCA and the largest ABC protein identified, consisting of 1,910 amino acids residues (Table 1). On the other hand, 6 half-size and 2 quarter-size ABCAs were found in tomato genome. A major feature of the ABCA subfamily is the presence of one AOH full-size ABCA in dicots, including tomato (Table 1), Arabidopsis [15], *L*. *japonicas* [18] and grape [19], that so far has not been identified in monocots, such as rice [16] and maize [17]. This suggests that the function of this full-size ABCA is specific to dicots.

The functions of ABCAs in plants are currently almost unknown, although mammalian ABCAs have been shown to be involved in numerous functions, such as lipid metabolism, cholesterol homeostasis, intracellular trafficking, pulmonary surfactant secretion and retinal transport [41]. AtABCA1 was reported to be related in pollen germination, seed germination and seed maturation [18,19]. Transcriptome analysis in Arabidopsis roots has revealed that *AtATH14* and *AtATH15* expressions are responsive to salt stress [42]. Among the 9 *SlABCAs*, ESTs of 5 members were available. The gene expression profiles from the eFP Browser revealed that *SlABCA1* and *SlABCA2* are preferentially expressed in the root (Table 1) and they might be involved in secretion activity of roots. *SlABCA4*-*7* are expressed specifically in the flower, suggesting a specific functions in floral organs (Table 1).

#### ABCB subfamily

The ABCB subfamily is the second largest subfamily. Full-size ABCBs are known as multidrug resistance protein (MDR) or P-glycoprotein (PGP) and the half-size ABCBs are characterized with names such as transporter associated with antigen processing (TAP), ABC transporter of mitochondria (ATM) and lipid A-like exporter putative (LLP) [35]. In the tomato genome, 29 members of this ABCB subfamily were identified and this comprises 18 full-size, 8 half-size and 3 quarter-size (Table 1) while in Arabidopsis, 22 full-size proteins, 6 half-size proteins and no quarter-size are identified. Surprisingly, according to the database, SlABCB18 contains 5 domains, i.e. TMD-TMD-NBD-TMD-NBD, and SlABCB19 contains 3 domains, i.e. NBD-TMD-NBD. These unique topological arrangements, i.e. additional TMDs or NBDs in their forward orientations maybe caused by a prediction error for the CDS or indicate that these sequences are pseudogenes (Table 1).

**Fig 3:**
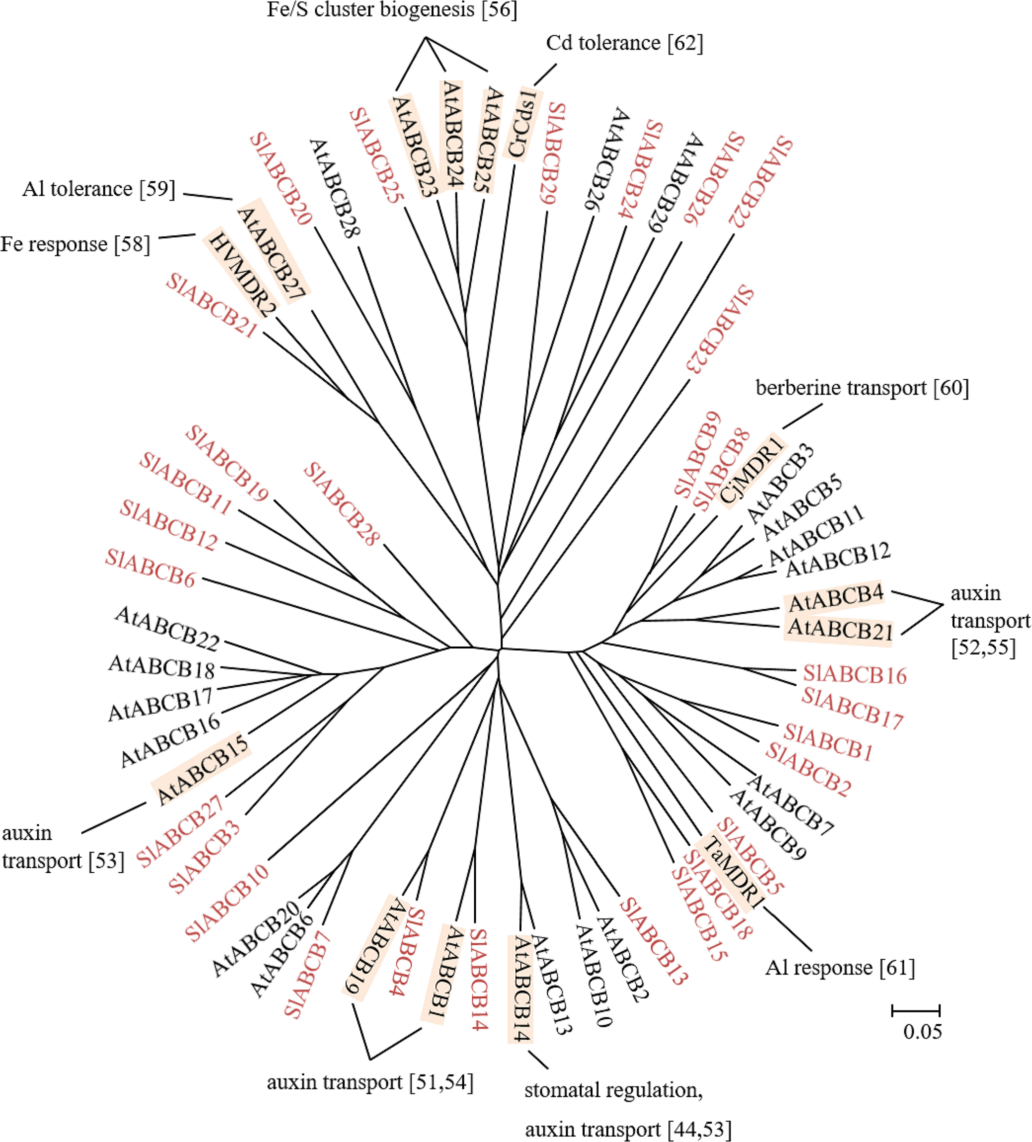
Phylogenetic tree of plant ABCB subfamily. ABCBs of tomato, Arabidopsis, barley (HvMDR2: BAC53613), wheat (TaMDR1: BAB85651), *Coptis japonica* (CjMDR1: BAB62040) and *Chlamydomonas reinhardtii* (CrCds1: AAQ19846) were subjected to phylogenetic analysis. Tomato ABCBs are shown in red. Physiological functions and references are indicated. The scale indicated in the figure shows 5% divergence between protein sequences.

All the characterized full-size ABCBs in Arabidopsis are localized to the plasma membrane [43,44], whereas the half-size ABCBs, ATMs (AtABCB23-25) have been reported to reside in mitochondria [45,46] while TAPs (AtABCB26 and AtABCB27) have been detected in the chloroplast [47] and vacuolar membrane [8,48]. In humans, ABCBs are associated with multi-drug resistance [36], lipid transport [49], iron and peptide transports [50]. Plant ABCBs are associated with several physiological functions as shown in Fig 3. For instance, AtABCB1 [51], AtABCB4 [52], AtABCB14, AtABCB15 [53], AtABCB19 [54] and AtABCB21 [55] are implicated in auxin transport in Arabidopsis. AtACBB14 was also reported to be associated with regulation of stomatal opening and closing [44]. AtABCB23, AtABCB24 and AtABCB25 modulate Fe-S cluster biogenesis [56]. AtABCB25 is involved in molybdenum cofactor biosynthesis and heavy metal tolerance, probably through their function as glutathione disulfide (GSSG) transporters [57]. AtABCB27 and its homologue in barley, HvMDR2 are responsible for Al and Fe sequestration respectively [58,59]. In *Coptis japonic*a, CjMDR1 transports berberine [60]. In wheat, TaMDR1 modulates aluminum toxicity responses and cadmium homeostasis [61]. In *Chlamydomonas reinhardtii*, CrCds1 mediates tolerance to cadmium [61,62].

In tomato, only 10 ESTs out of 29 the SlABCBs were available (Table 1, Fig 3). Based on the eFP Browser gene expression data, *SIABCB7*, *SIABCB13*, *SIABCB14*, *SIABCB18*, *SIABCB20*, *SIABCB21*, *SIABCB24*, *SIABCB25* and *SIABCB29* are ubiquitously expressed in all organs and tissues (Table 1), suggesting their responsibilities for basic cellular maintenance. Most of *SlABCBs* are highly expressed in the root. This may suggest an involvements of these SlABCBs in ion and heavy metal transports in roots.

#### ABCC subfamily

ABCCs are also called multidrug resistance-associated proteins (MRP) due to their function in transporting glutathione- and glucuronide-conjugates in drug-resistant animal cancer cells [35]. In plants, full-size ABCCs were earlier characterized and later half-size ABCCs were found in Arabidopsis and rice genomes and characterized [4,17]. In plants, most ABCCs are characterized as vacuolar localized proteins and few have been reported to reside on the plasma membrane [17]. Maize ZmMRP3 and grape VvABCC1 are involved in anthocyanin accumulation in vacuoles [6,7]. Arabidopsis AtABCC1-4 and wheat TaMRP1 are involved in transport of glutathione-conjugates [63]. Arabidopsis AtABCC5 [64], maize ZmMRP4 [65] and rice OsABCC13 [66] are implicated in phytate transport [67]. AtABCC2 and AtABCC3 are involved in chlorophyll catabolite transport [63]. AtABCC1 and AtABCC4 are implicated in folate transport [63]. AtABCC4 and AtABCC5 are functionally related to stomatal regulation [63]. AtABCC3, AtABCC6 and AtABCC7 confer heavy metal resistance [68,69].

**Fig 4:**
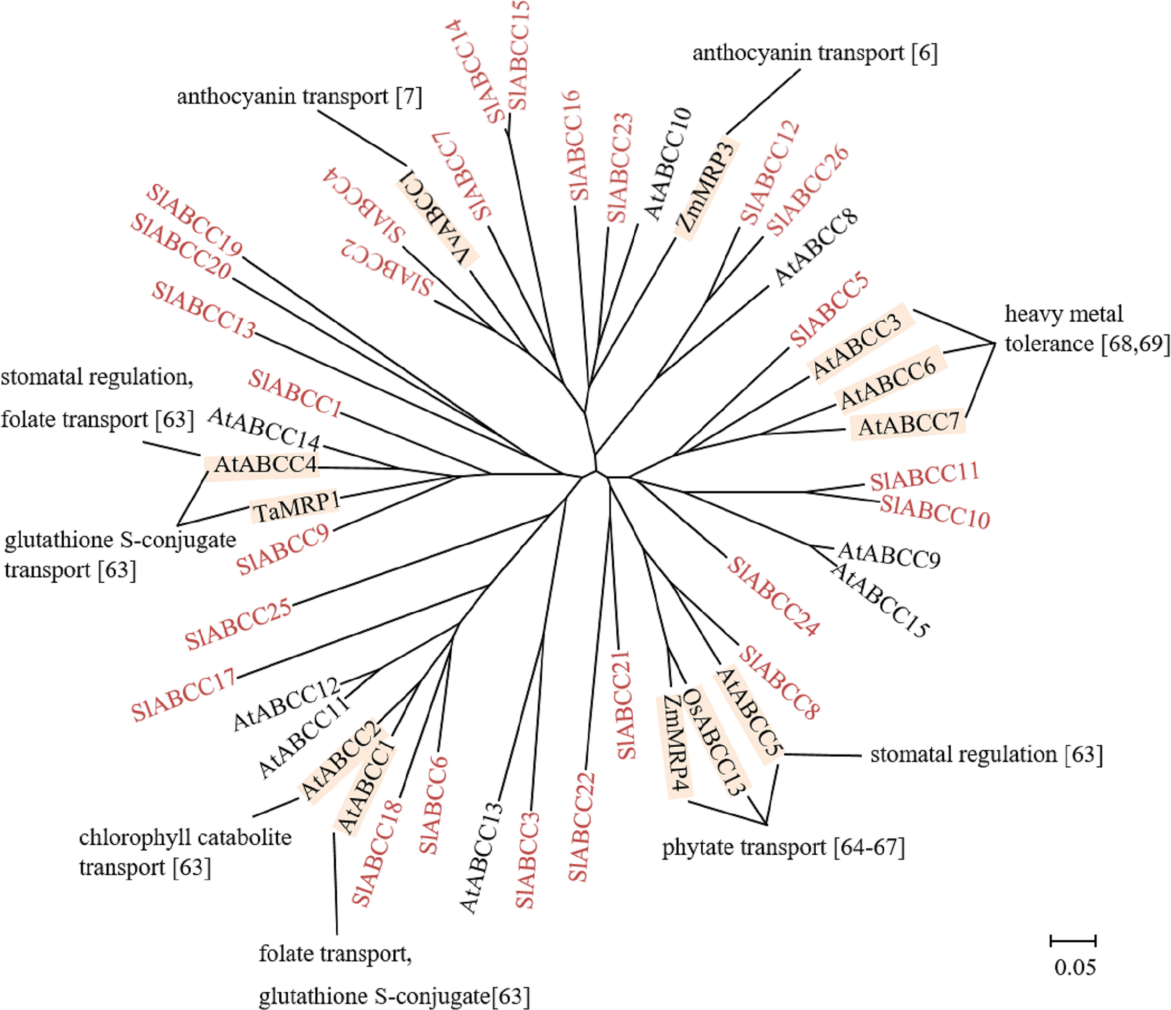
Phylogenetic tree of plant ABCC subfamily. ABCCs of tomato, Arabidopsis, rice (OsABCC13: *Os03g0142800*), maize (ZmMRP3: AAT37905, ZmMRP4: ABS81429), wheat (TaMRP1: AAL47686) and grape (VvABCC1: AGC23330) were subjected to phylogenetic analysis. Tomato ABCCs are shown in red. Physiological functions and references are indicated. The scale indicated in the figure shows 5% divergence between protein sequences.

In the tomato genome, 26 members of the ABCC subfamily were found and this comprises 12 full-size, 6 half-size and 8 quarter-size ABCCs. SlABCC13 shows a unique protein structure, i.e. TMD–NBD–TMD (Table 1, Fig 4), however as for the non-typical ABCBs this might reflect a prediction error for the CDS or the presence of a pseudogene. SlABCC18 shows reverse orientation (NBD-TMD), which is different from other SlABCCs (TMD–NBD). ESTs for 11 ABCCs were available (Table 1). The gene expression profile of the tomato eFP Browser shows that *SlABCC1*, *SlABCC7*, *SlABCC10*, *SlABCC11*, *SlABCC13*, *SlABCC19*, *SlABCC20* and *SlABCC21* are preferentially expressed in the later stages of fruit development (Table 1). These *SlABCCs* might play important roles in fruit ripening, such as chlorophyll degradation and secondary metabolite accumulation in the vacuole.

#### ABCD subfamily

**Fig 5:**
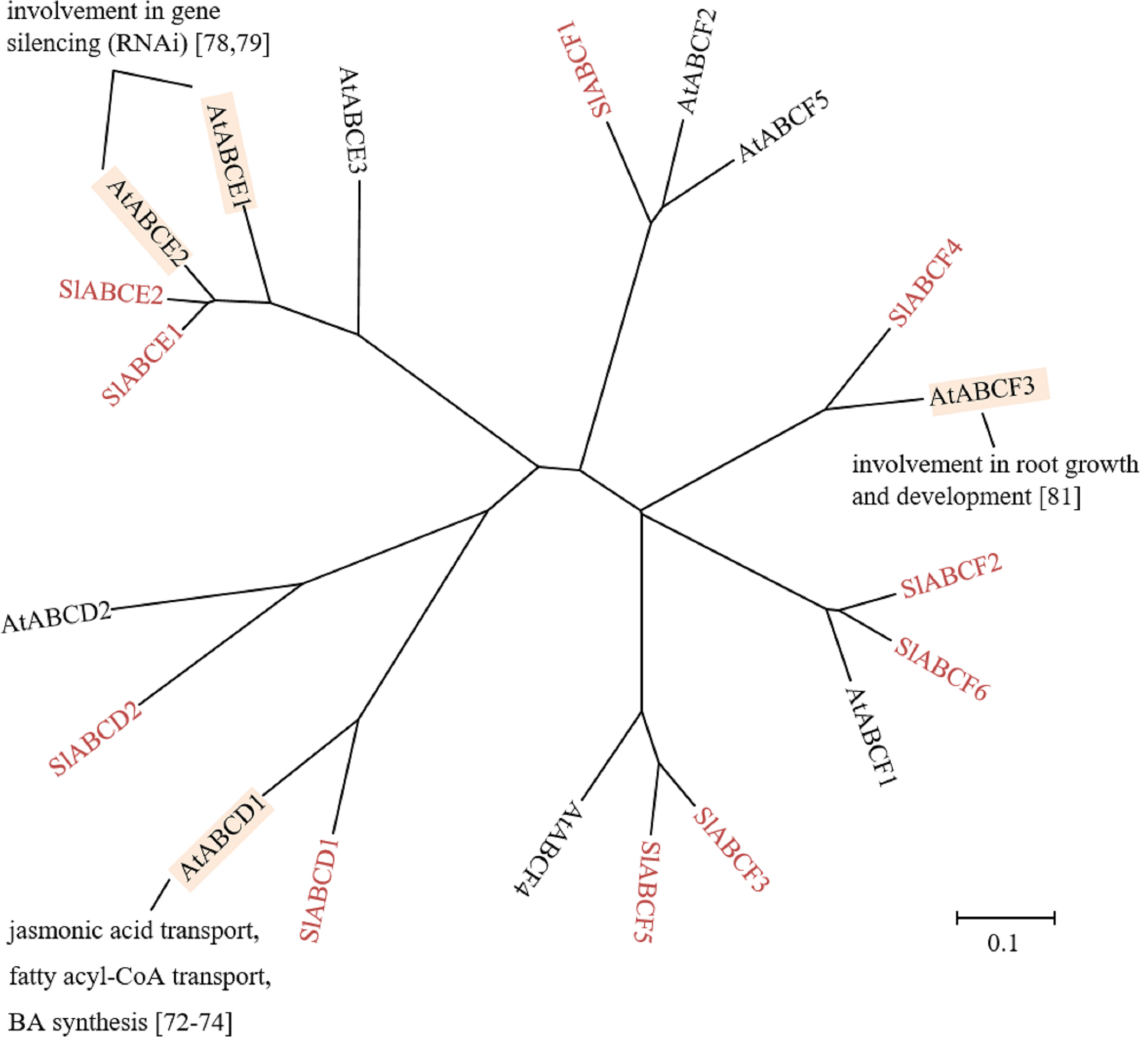
Phylogenetic tree of plant ABCD, ABCE and ABCF subfamilies. ABCDs, ABCEs and ABCFs of tomato and Arabidopsis were subjected to phylogenetic analysis. Tomato ABC proteins are shown in red. Physiological functions and references are indicated. The scale indicated in the figure shows 10% divergence between protein sequences.

ABCDs are also known as peroxisomal membrane proteins (PMPs) and are localized in the peroxisomal membrane [70,71]. In humans, they are exclusively known to be half-size proteins with TMD-NBD orientation, whereas, in plants, both half- and full-size ABC proteins exist [15]. AtABCD1 is implicated in benzoic (BA) synthesis [72], transport of 12-oxophytodienoic acid (OPDA) [73] and jasmonic acids (JA) [74]. The *AtABCD1* mutant is impaired in seed germination [75] and fertility [76]. The tomato genome contains one full-size and one half size ABCDs were found (Table 1, Fig 5). The gene expression profile of the tomato eFP Browser shows constitutive gene expression of both *SlABCDs* (Table 1). It is likely that these transporters exhibit similar functions as their Arabidopsis counterparts and that they are involved in peroxisomal import of long chain fatty acids.

#### ABCE subfamily

ABCEs, also called RNase L inhibitor (RLI), possess an N-terminal Fe-S domain, which interacts with nucleic acids [30]. All ABCE subfamily members are soluble ABC proteins harboring two conserved NBDs (NBD-NBD) [17]. In humans, only one ABCE exists and it is involved in ribosome biogenesis and control of translation [77]. There are 3 ABCEs present in Arabidopsis and two each in rice [16], maize [17], grape [19], *L*. *japonicas* [18], *H*. *brasiliensis* [4] and also in tomato (Table 1, Fig 5). In Arabidopsis, AtABCE1 and ABCE2 are involved in RNA interference (RNAi) regulation [78,79]. Among the two tomato *SlABCEs*, only one EST of *SlABCE1* was available (Table 1). The tomato eFP Browser revealed that both *SlABCE1* and *SlABCE2* are expressed constitutively in all organs and tissues (Table 1) and may play roles in ribosome biogenesis, control of translation and gene silencing regulation.

#### ABCF subfamily

ABCFs are also called general control non-repressible homologs (GCN). The ABCF subfamily is similar to the ABCE subfamily [17], because ABCFs are also soluble ABC proteins containing two fused NBDs (NBD-NBD). In yeast and humans, ABCFs are involved in gene expression regulation [16,80]. In Arabidopsis, 5 ABCFs are present and AtABCF3 is implicated in root growth [81]. In tomato, 6 ABCFs were identified and ESTs were available for 5 ABCFs (Table 1, Fig 5). The Tomato eFP Browser showed constitutive expressions for all 6 *SlABCFs* (Table 1).

#### ABCG subfamily

The ABCG subfamily is the largest subfamily in plants while only 5 ABCGs are present in humans [17]. The ABCG subfamily is made up of full-size and half-size ABC proteins, also called pleiotropic drug resistance (PDR) or white-brown complex (WBC), respectively [35]. All full-size and half-size ABCGs have two, respectively one NBD-TMD, respectively, and function as ABC transporters. In the tomato genome, 70 ABCGs were found, which are made up of 22 full-size, 32 half-size and 16 quarter-size ABC proteins (Table 1). This number is larger than the 44 ABCGs reported for Arabidopsis [15]. In humans, ABCGs function as transporters of cholesterol, urate, haem, and other pharmaceutical compounds [82]. On the other hand, in plants, ABCGs have been reported to transport various phytohormones, including abscisic acid (ABA), cytokinin, strigolactone and auxin derivatives [10].

**Fig 6:**
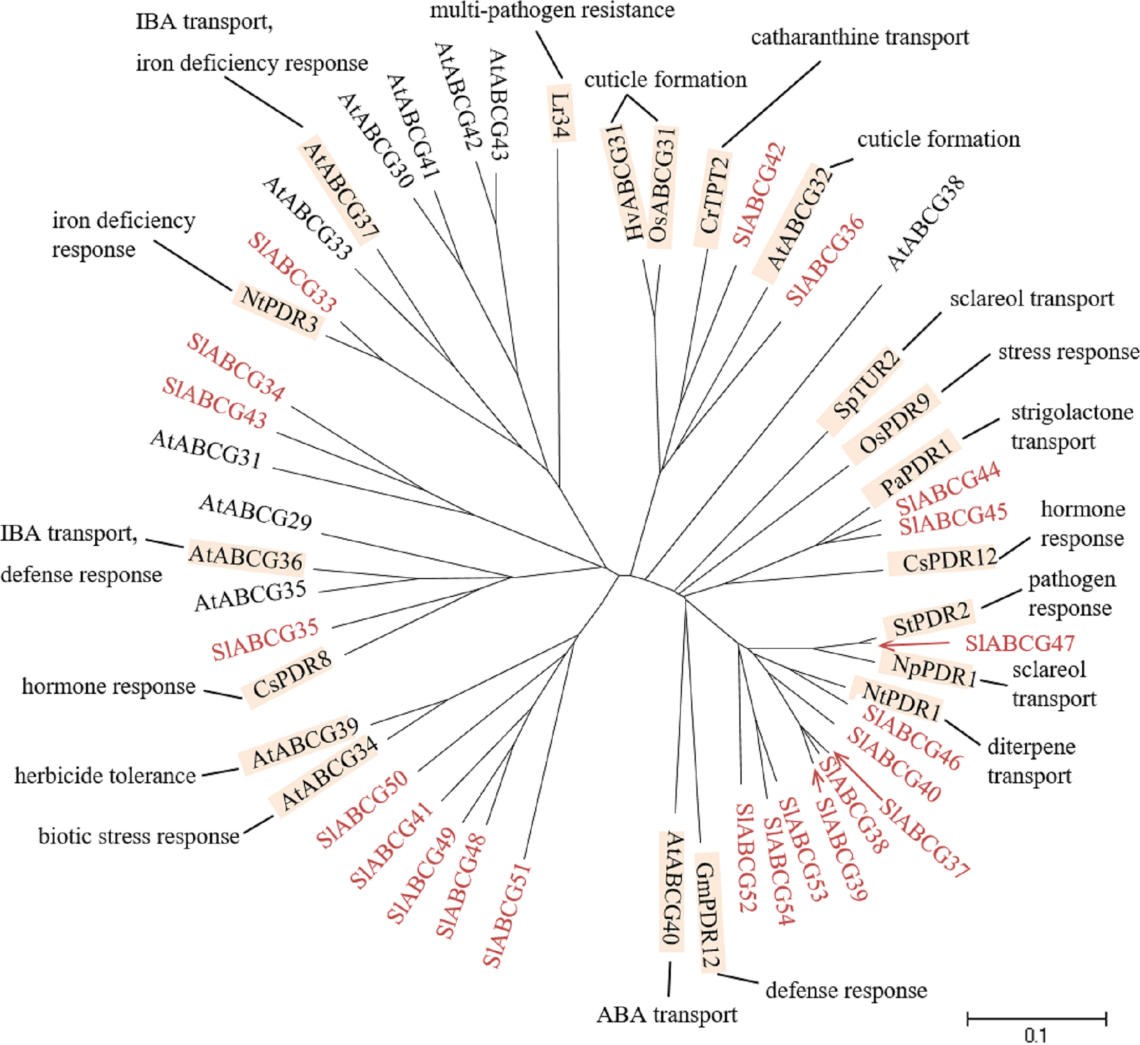
Phylogenetic tree of plant full-size ABCGs. ABCGs of tomato, Arabidopsis, rice (OsABCG31: *Os01g0177900*, OsPDR9: *Os01g0609300*), wheat (Lr34: ACN41354), barley (HvABCG31: NP_001237697), soybean (GmPDR12: NP_001237697), cucumber (CsPDR8: ACU82514, CsPDR12: ACU82515), *Nicotiana plumbaginifolia* (NpPDR1: Q949G3, NpPDR2: CAH40786), *N*. *tabacum* (NtPDR1: AGN95757, NtPDR3: CAH39853), petunia (PaPDR1: AFA43816), potato (StPDR2: AEB65936), periwinkle (CrTPT2: KC511771) and duckweed (SpTUR2: CAA94437) were subjected to phylogenetic. Tomato ABCGs are shown in red. Physiological functions and references are indicated. Details on the functions are reviewed in [83,84]. The scale indicated in the figure shows 10% divergence between protein sequences.

One of the most widely studied ABC protein subfamily in plants are the full-size ABCGs, also called PDRs. A detailed review on plant full-size ABCGs is available [83,84] and a highlight on their functions is shown in Fig 6. The subcellular localization of full-size ABCGs is the plasma membrane [84]. Full-size ABCGs of Arabidopsis AtABCG32 [85], rice OsABCG31 [86], barley HvABCG31 [86] are involved in cuticle formation. The *N*. *plumbaginifolia* NpPDR1 [87] and duckweed SpTUR2 are known to participate in sclareol transport [88].

**Fig 7:**
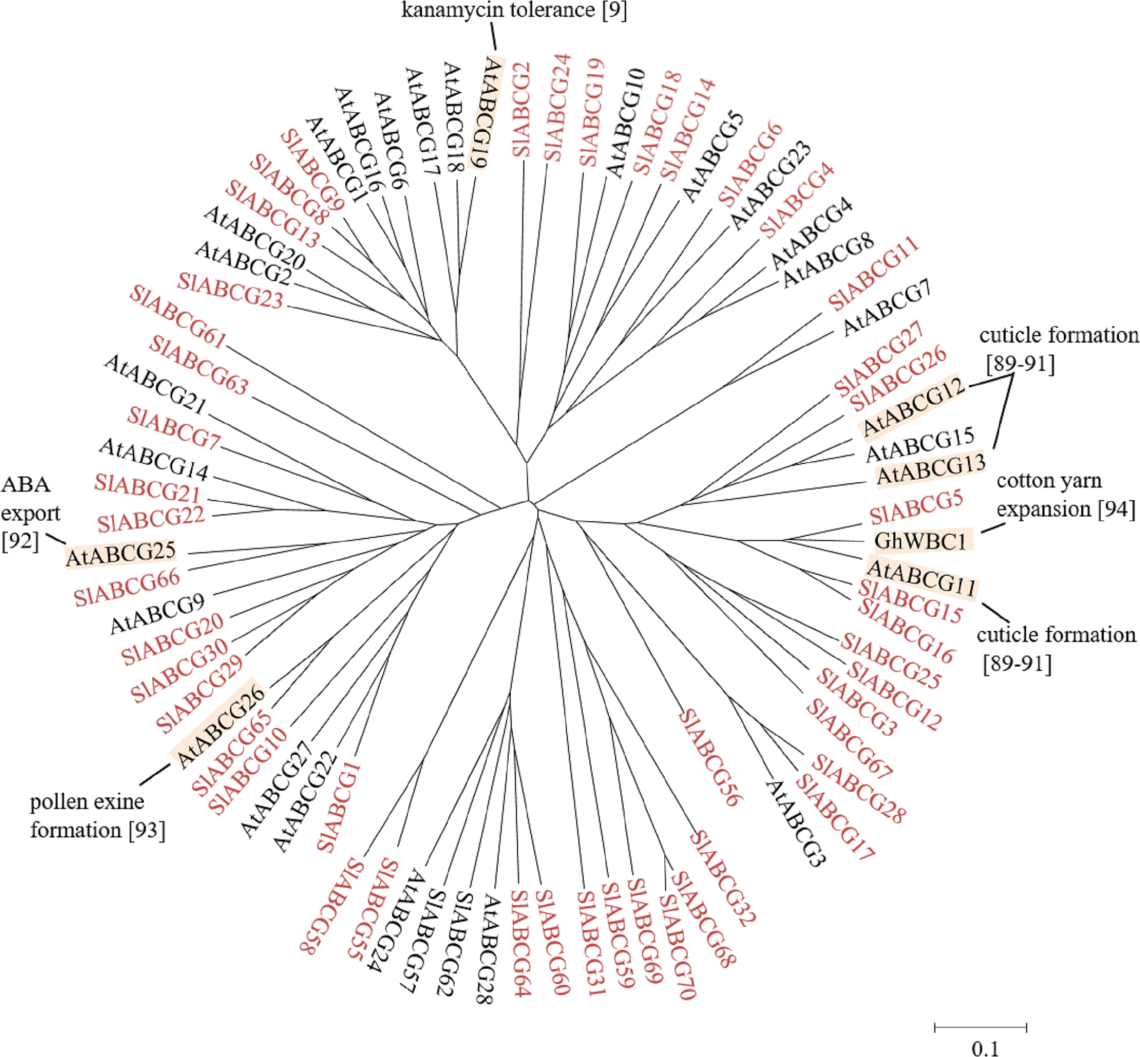
Phylogenetic tree of plant half-size ABCGs. ABCGs of tomato, Arabidopsis and cotton (GhWBC1: AAP80385) were subjected to phylogenetic analysis phylogenetic analysis. Tomato ABCGs are shown in red. Physiological functions and references are indicated. The scale indicated in the figure shows 10% divergence between protein sequences.

Half-size ABCGs are also called WBCs, have been reported to be localized in the plasma membrane, mitochondrial membrane, chloroplast membrane and cytoplasm [17]. The physiological roles of half-size ABCGs are summarized in Fig 7. In Arabidopsis, half-size ABCGs, i.e. AtABCG11-13 are implicated in cuticle formation [89–91]. On the other hand, AtABCG19 confers kanamycin resistance [9]. AtABCG25 has been reported to act as an ABA exporter [92] and AtABCG26 is involved in pollen development [93]. In cotton, GhWBC1 is involved in cotton yarn expansion [94].

The tomato eFP browser shows specific expressions of *SlABCG12*, *SlABCG16*, *SlABCG31*, *SlABCG32*, *SlABCG44*, *SlABCG45*, *SlABCG51*, *SlABCG52*, *SlABCG55* and *SlABCG58* (Table 1), suggesting their importance in root. *SlABCG25*, *SlABCG27*, *SlABCG29*, *SlABCG30*, *SlABCG43*, *SlABCG65*, *SlABCG68* and *SlABCG70* are expressed specifically in bud. Interestingly, only *SlABCG*59, which encodes a quarter-size ABCG, shows specific expression in mature fruit, although other *SlABCG*s are also expressed in fruits. Although we cannot guess the function of SlABCG59, it may play an important roles in tomato fruit maturation.

#### ABCI subfamily

**Fig 8:**
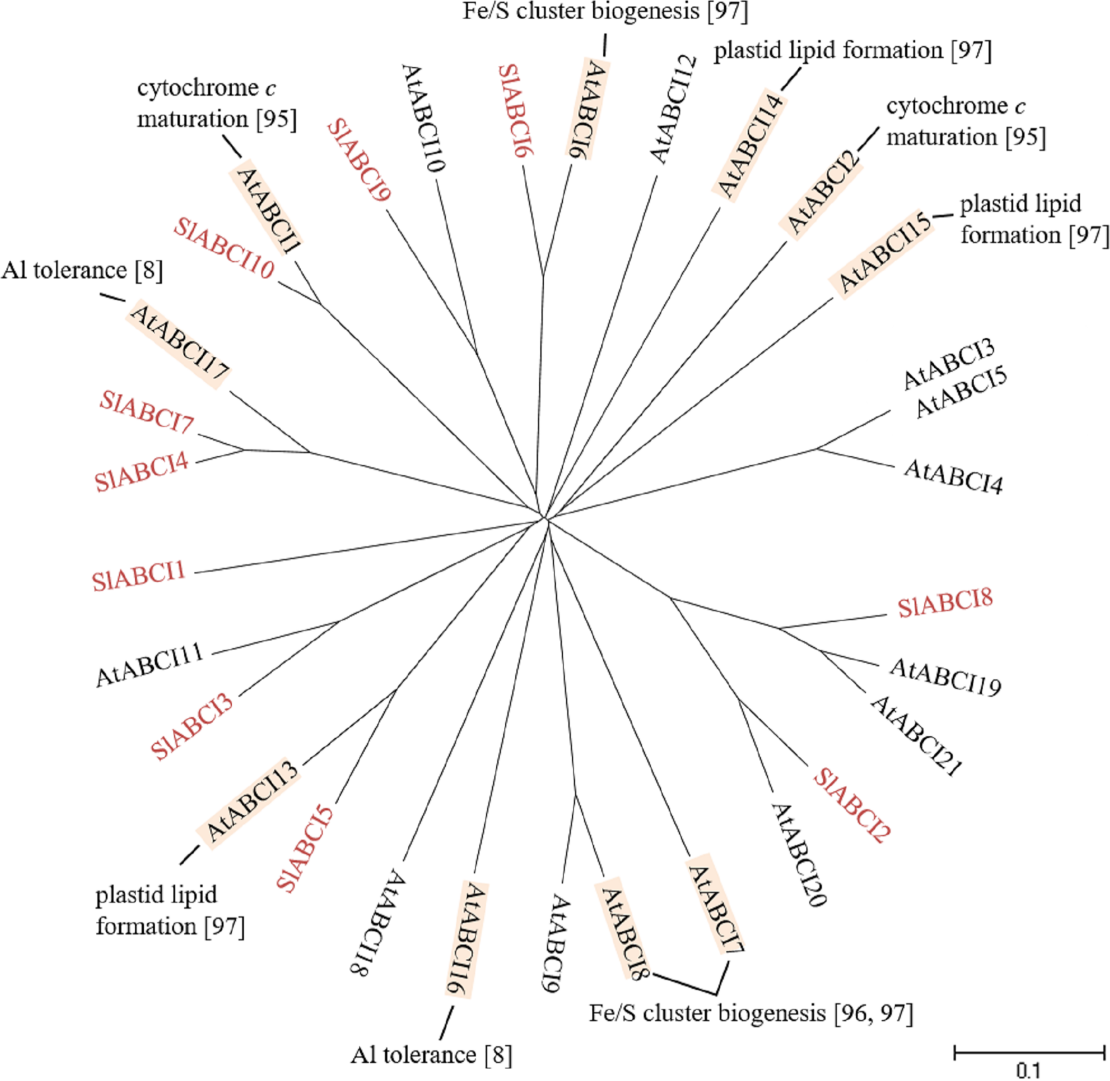
Phylogenetic tree of plant ABCI subfamily. ABCIs of tomato and Arabidopsis were subjected to phylogenetic analysis. Tomato ABCIs are shown in red. Physiological functions and references are indicated. The scale indicated in the figure shows 10% divergence between protein sequences.

ABCIs are also called non-intrinsic ABC proteins (NAPs). ABCIs are soluble ABC proteins possessing a single ATP binding domain [35]. In Arabidopsis, AtABCI1 and AtABCI2 are reported to be involved in cytochrome c maturation (CCM) [95]. AtABCI6-8 are implicated in biosynthesis of Fe/S cluster [96,97]. AtABCI13-15 are responsible for plastid lipid formation [97]. On the other hand, AtABCI16 and AtABCI17 confer tolerance to aluminum [8]. In the tomato genome, 10 *SlABCIs* have been identified and ESTs for 8 *SlABCIs* were available (Table 1, Fig 8). The gene expression profiles from the tomato eFP Browser showed that *SlABCI4*, *SlABC16* and *SlABC18* are constitutively expressed in roots and floral organs, respectively, and *SlABCI5*, *SlABCI6*, *SlABCI9 and SlABCI10* in developing fruits (Table 1), suggesting their specific functions in these organs and tissues.

### Gene expression analysis

**Fig 9:**
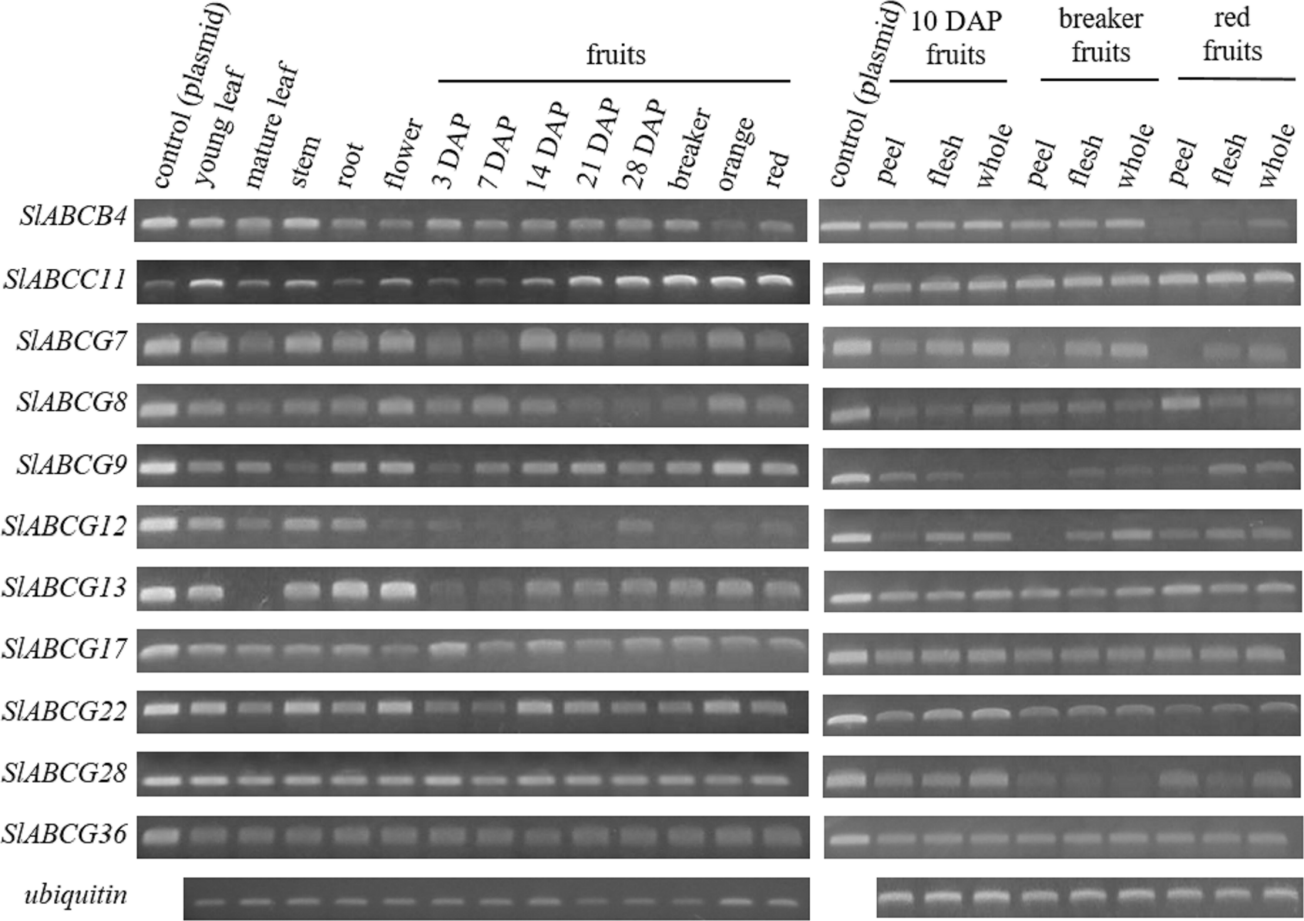
Gene expression analysis of selected ABC transporters in various tomato organs and tissues. RT-sqPCR analysis for selected tomato ABC transporters was performed using RNA extracted from the indicated organ or tissue and gene-specific primers (amplicons *~* 200 bp). Respective cDNA-containing plasmid was used as control. The ubiquitin gene was used as a constitutively expressed control gene. DAP:  days after pollination.

We chose SlABCB4, SlABCC11, SlABCG7, SlABCG8, SlABCG9, SlABCG12, SlABCG13, SlABCG17, SlABCG22, SlABCG28 and SlABCG36 for further gene expression analysis by RT-sqPCR (Fig 9). These genes were chosen because their full length cDNA sequences were available in TOMATOMICS database (http://plantomics.mind.meiji.ac.jp/tomatomics/). Therefore, we requested for their full length cDNA clones from National Bioresource Project (NBRP)-Tomato (http://tomato.nbrp.jp/indexEn.html) to sequence and then performed RT-sqPCR to identify their expression patterns.

Gene expression was detected in various organs of ‘MicroTom’, i.e. leaf, stem, root, flower and developing fruits. In addition, to obtain a detailed gene expression profile in fruits, gene expressions in fruit peel and flesh at 10 DAP, breaker and red stages were investigated. Although most *SlABCs* were ubiquitous expressed, some *SlABCs* exhibited a characteristic gene expression patterns (Fig 9).

*SlABCB4* showed ubiquitous expression, but its transcript level was lower in mature fruits (Fig 9). The closest orthologue of SlABCB4 in Arabidopsis is AtACB19, and has been reported to transports auxin [98]. This suggests that SlABCB4 might be responsible for auxin transport in various organs of tomato. *SlABCC11* expression was high in mature leaf and fruits after 21 DAP (Fig 9). Although the function of SlABCC11 is unclear because no close orthologue of Arabidopsis exists (Fig 3), it may play important roles in the later part of tomato fruit development.

Functions of half-size SlABCGs, SlABCG7, SlABCG8, SlABCG9, SlABCG12, SlABCG13, SlABCG17, SlABCG22 and SlABCG28 are unclear, because no characterized orthologue exists (Fig 7). *SlABCG7*, *SlABCG8*, *SlABCG9*, *SlABCG12*, *SlABCG13*, *SlABCG17*, *SlABCG22* and *SlABCG28* showed different expression patterns and *SlABCG9*, *SlABCG13*, *SlABCG17*, *SlABCG22* and *SlABCG28* showed relatively higher expression levels in fruits (Fig 9), suggesting that they may play some their roles in fruit development and/or ripening.

*SlABCG36*, which encode a full-size SlABCG, showed ubiquitous expression in all organs (Fig 9). SlABCG36 is likely to transport metabolites involved in cuticle formation, because its closest orthologue of Arabidopsis, AtABCG32 is responsible for cuticle formation (Fig 6) [85]. Therefore we expected high *SlABCG36* expression in fruit peel. However, the differences in *SlABCG36* expressions between in fruit peel and flesh were not pronounced, although it was slightly higher in the peel than in flesh of red fruit (Fig 9).

## Conclusion

This study revealed the presence of 154 putative ABC proteins in the tomato genome. Based on the phylogenetic analysis, the ABC proteins were grouped into their respective subfamilies, ABCA through to ABCI, except ABCH. Members of ABCG, ABCB and ABCC subfamilies were the most abundant, whiles ABCD and ABCE subfamilies were less abundant. Among the 154 tomato ABC proteins, 47 members are soluble ABC proteins, while 107 members encode for ABC transporters with TMDs. As far as we know, this study is the only genome-wide analysis of ABC proteins in the Solanaceae species. In this study, we provided the fundamental and exhaustive information about tomato ABC proteins, i.e. the list of all ABC proteins in tomato with their locus numbers (gene IDs), protein topology, best hit ESTs, gene expression data (Table 1) and phylogenetic trees of subfamily members and orthologues in other plants, showing the reported physiological functions (Figs 2–8). This information is indispensable for further studies of ABC proteins not only in tomato but also in other Solanaceae species. We hope this study will be useful to many researchers studying plant ABC proteins.

## Supporting information

S1 Table Primers and PCR conditions for RT-sqPCR. The forward and reverse primers, PCR condition and number of PCR cycles for each ABC transporter or control gene (*ubiquitin*) are shown.
(DOCX)

S2 Table Comparison of non-overlapping tomato ABC proteins in this study and in Andolfo et al. 2015 and presence of nucleotide binding domain (NBD). The presence of nucleotide binding domain (NBD) was confirmed using Pfam web server (http://pfam.xfam.org/).
(XLSX)

S3 Table Comparison of genes putatively encoding ABC proteins in two tomato genome databases, SL3.0 and ITAG3.10 from Sol Genomics Network and TMCSv1.2.1 from TOMATOMICS. Genes putatively encoding ABC proteins in TMCSv1.2.1 from TOMATOMICS (http://plantomics.mind.meiji.ac.jp/tomatomics/download.php) were obtained by blasting using the protein sequences (Table 1) from SL3.0 and ITAG3.10 of Sol Genomics Network (https://solgenomics.net/organism/Solanum_lycopersicum/genome). Identical genes between two different tomato genome databases and splicing variants were confirmed by comparing their positions in chromosome.
(XLSX)
