# Supplementary material for: Genome-wide analysis of ATP binding cassette (ABC) transporters in tomato
Source: PLoS One. 2018 Jul 26;13(7):e0200854. doi: 10.1371/journal.pone.0200854 (PMC6062036; doi:10.1371/journal.pone.0200854)
Supplement: S1 Table — The forward and reverse primers, PCR condition and number of PCR cycles for each ABC transporter or control gene (ubiquitin) are shown. (DOCX) [file pone.0200854.s001.docx]

**S2 Table. Primers and PCR conditions for RT-sqPCR.**

| *SlABCB4* | Primers | Forward | 5’- GCAATCAACATGGCCTAGTG -3’ |
| --- | --- | --- | --- |
|  |  | Reverse | 5’- AAAGTCACTAGGACCCAAACA -3’ |
|  | Condition | 98℃ 10s, 55℃ 30s, 72℃ 10s | |
|  | Number of cycles | Organs and fruit development stages: 35 | |
|  |  | Peel, fresh and whole fruit: 35 | |
| *SlABCC11* | Primers | Forward | 5’- CATTCTGCAAAAAGCAGTGAA -3’ |
|  |  | Reverse | 5’- AAAGTTCCAATGCTGGGATG -3’ |
|  | Condition | 98℃ 10s, 55℃ 30s, 72℃ 10s | |
|  | Number of cycles | Organs and fruit development stages:　40 | |
|  |  | Peel, fresh and whole fruit:　40 | |
| *SlABCG7* | Primers | Forward | 5’- TGGTGGCATTGGTTGTTATG -3’ |
|  |  | Reverse | 5’- CACTTTAGTCATTGATGCCAC -3’ |
|  | Condition | 98℃ 10s, 55℃ 30s, 72℃ 10 s | |
|  | Number of cycles | Organs and fruit development stages:　35 | |
|  |  | Peel, fresh and whole fruit:　35 | |
| *SlABCG8* | Primers | Forward | 5’- GGTTTTATAAATTTGGGTTTTAGGTG -3’ |
|  |  | Reverse | 5’- TTCAAGCCATGAAATTATAGCC -3’ |
|  | Condition | 98℃ 10 s, 55℃ 30 s, 72℃ 10 s | |
|  | Number of cycles | Organs and fruit development stages:　37 | |
|  |  | Peel, fresh and whole fruit:　37 | |
| *SlABCG9* | Primers | Forward | 5’- CAAACCGTGAAAGCTCTTGAA -3’ |
|  |  | Reverse | 5’- CTACCGTCTGTCTCCGATCA -3’ |
|  | Condition | 98℃ 10 s, 55℃ 30 s, 72℃ 10 s | |
|  | Number of cycles | Organs and fruit development stages:　37 | |
|  |  | Peel, fresh and whole fruit:　37 | |
| *SlABCG12* | Primers | Forward | 5’- CCTCGTCGGATTACAAAAGG -3’ |
|  |  | Reverse | 5’- CAGGGACAATGCTTGCTAC -3’ |
|  | Condition | 98℃ 10 s, 55℃ 30 s, 72℃ 10 s | |
|  | Number of cycles | Organs and fruit development stages:　34 | |
|  |  | Peel, fresh and whole fruit:　37 | |
| *SlABCG13* | Primers | Forward | 5’- TCGTGTGATCAATCAGAGGTG -3’ |
|  |  | Reverse | 5’- CAATTCAAACACAACCCTTGG -3’ |
|  | Condition | 98℃ 10 s, 55℃ 30 s, 72℃ 10 s | |
|  | Number of cycles | Organs and fruit development stages:　37 | |
|  |  | Peel, fresh and whole fruit:　40 | |
| *SlABCG17* | Primers | Forward | 5’- TCGATCCAGGGTTGGTAAAC -3’ |
|  |  | Reverse | 5’- CTGAACTGAAGGGTCACAAGG -3’ |
|  | Condition | 98℃ 10 s, 64℃ 30 s, 72℃ 10 s | |
|  | Number of cycles | Organs and fruit development stages:　40 | |
|  |  | Peel, fresh and whole fruit:　40 | |
|  |  |  | |
|  |  |  |  |
| *SlABCG22* | Primers | Froward | 5’- GGGAACCATTTCAAGAGGAA -3’ |
|  |  | Reverse | 5’- GGTAAAACATTGCCAGAGTG -3’ |
|  | Condition | 98℃ 10 s, 63℃ 30 s, 72℃ 10 s | |
|  | Number of cycles | Organs and fruit development stages:　40 | |
|  |  | Peel, fresh and whole fruit:　37 | |
| *SlABCG28* | Primers | Forward | 5’- GGTTTTTCAGTGAGCAACAC -3’ |
|  |  | Reverse | 5’- CACTTACAAATCACTGGATCTG -3’ |
|  | Condition | 98℃ 10 s, 63℃ 30 s, 72℃ 10 s | |
|  | Number of cycles | Organs and fruit development stages:　42 | |
|  |  | Peel, fresh and whole fruit:　42 | |
| *SlABCG36* | Primers | Forward | 5’- TTGCAGAATGGAGTA AATTTGG -3’ |
|  |  | Reverse | 5’- TGACGCCCTAATCACTTTTG -3’ |
|  | Condition | 98℃ 10s, 55℃ 30s, 72℃ 10s | |
|  | Number of cycles | Organs and fruit development stages:　38 | |
|  |  | Peel, fresh and whole fruit:　38 | |
| *ubiquitin* | Primers | Forward | 5’- CACCAAGCCAAAGAAGATCA -3’ |
|  |  | Reverse | 5’- TCAGCATTAGGGCACTCCTT -3’ |
|  | Condition | 95℃ 5 s, 60℃ 30 s | |
|  | Number of cycles | 30 | |

The forward and reverse primers, PCR condition and the number of PCR cycles for each ABC transporter genes or control gene (*ubiquitin*) are shown.
